# Identification and antimicrobial susceptibility profiles of *Staphylococcus* species isolated from raw cow milk, and swabs in smallholder dairy farms in Meta district, Eastern Ethiopia

**DOI:** 10.1186/s12866-024-03439-6

**Published:** 2024-08-01

**Authors:** Abrahim Dawed Ahmed, Adem Hiko, Dinaol Belina, Haben Fesseha Gebremeskel, Isayas Asefa Kebede

**Affiliations:** 1 Eastern Branch, Ethiopian Agriculture Authority, Dire Dawa, Ethiopia; 2 College of Veterinary Medicine, Haramaya University, P. O. Box 138, Dire Dawa, Ethiopia; 3 School of Veterinary Medicine, Wolaita Sodo University, Wolaita Sodo, Ethiopia; 4 School of Veterinary Medicine, Ambo University, P. O. Box 19, Guder, Ethiopia

**Keywords:** Hygiene, Staphylococcus aureus, Antimicrobial resistance, Raw milk, Meta, Ethiopia

## Abstract

**Background:**

The safety of milk production in terms of foodborne infections is a worldwide issue, particularly in developing countries where production is often unhygienic. A cross-sectional study was conducted from December 2018 to August 2019 in the Meta District of Eastern Hararghe Zone, Oromia Regional State, Ethiopia. We aim to assess milk hygiene practices among smallholder dairy farmers, estimate the prevalence of *Staphylococcus aureus* in raw cow milk and swabs, assess associated risk factors, and the antimicrobial susceptibility test of *S. aureus* isolates. Face-to-face interviews with 30 respondents randomly selected from smallholder dairy farmers were used to assess the potential risk factors for *S. aureus* contaminations in milk. A total of 177 samples were examined using standard microbiological testing. The disc diffusion technique was also employed to assess the antibiotic susceptibility of the isolates. The data was analyzed using STATA^®^ version 14.0 statistical software.

**Results:**

According to the milk hygiene assessment, 80% of respondents did not wash cow udder before milking, did not use detergent to clean milk containers, and did not keep milk refrigerated before consumption or sale, while 63.3% of milk consumers ingested raw milk. They had never heard of staphylococci foodborne disease. Likewise, the overall prevalence of *S. aureus* was 12.42% (95%CI: 8.32–18.98). The prevalence of *S. aureus* in udder milk, equipment swabs, and milkers’ hands was 18.8%, 26.7%, and 30%, respectively. The prevalence of *S. aureus* in milk is significantly associated with age, and mastitis history (*p* < 0.05). Moreover, old and mastitis positive animals were eight (OR: 8.40; 95%CI: 1.68–41.89) and four (OR: 4.33; 95%CI: 1.37–13.66) times more likely to be infected by *S. aureus* than adult, and mastitis negative animal. The isolates were resistant to penicillin G (97.4%) and tetracycline (69.2%) whereas susceptible to kanamycin, streptomycin, vancomycin, and cefotaxime, at 84.6%, 71.8%, 64%, and 58.8%, respectively.

**Conclusion:**

This study revealed the presence of antimicrobial-resistant patterns of *S. aureus* on commonly used antibiotics, as well as inadequate milk handling practices in the study area. Thus, awareness should be created on proper milk handling and hygiene as well as appropriate uses of antibiotics should be encouraged.

**Supplementary Information:**

The online version contains supplementary material available at 10.1186/s12866-024-03439-6.

## Background

Global population expansion and lifestyle changes have increased demand for high-quality animal-derived foods, while the number of food poisoning cases is growing worldwide. Ensuring food safety to protect public health and promote economic growth, on the other hand, is a major challenge for both developing and developed countries [1].

Foodborne infections are one of the most common public health issues in the globe. Food contamination during manufacturing, collection, transportation, and preparation or processing can all cause human disease [2]. Foodborne infections are estimated to result in 600 million cases and 420,000 deaths worldwide. Bacteria are usually blamed for foodborne diseases [3].

Milk is an important source of nutrition for both humans and animals, and it is believed to be the first and sole diet for mammals’ newborns since it is nearly complete [4, 5]. Milk for human consumption must be pathogen-free. Microbial contamination in milk has been connected to human ailments, as well as milk degradation. Many milk-borne epidemics of human diseases are caused by milk contamination [6]. Primary microbial contamination in milk may be caused by a diseased lactating animal. Secondary sources of microbial contamination include milkers’, milk handlers, uncleaned utensils, and/or milking equipment, as well as water supplies used for clean purposes [7].

The issue of milk safety is widespread [8]. This is particularly true in underdeveloped countries such as Ethiopia, where raw milk and other dairy products are frequently produced and consumed in unhygienic conditions [9]. The safety of raw milk and raw milk products in terms of staphylococcal poisoning is a major global concern. When the mammary gland is infected, milk can become contaminated with *Staphylococcus aureus*. Furthermore, improper hygiene habits, such as not washing your hands thoroughly when handling milking equipment and coughing or sneezing, might contaminate it during or after milking. In this situation, human activity is to blame for the contamination because these germs infiltrate human nasal passageways. Improper storage and preparation settings, as well as dirty utensils, contribute to raw food contamination [10].

The dairy industry is a major food sector in many countries across the world, and it has mainly been successful in creating safe products. Despite this, public health officials remain concerned about the items’ safety. Milk is particularly nutrient-dense and provides an ideal environment for the growth of many microorganisms; contamination of these products can occur at various points in the food chain via frequently complex pathways; and these products have been the source of food-borne infections caused by a variety of microbial and chemical hazards [11]. Staphylococci are bacteria that can be found in the skin and mucous membranes of both animals and humans [10]. They are also widespread and have been found in a variety of environments, including air, water, soil, and plant surfaces, as well as meat, poultry, and dairy products [10]. Pathogenic strains are frequently coagulase-positive and have been demonstrated to cause sickness in hosts all over the world [12]. They can cause mild to severe diseases, including foodborne illnesses. *S. aureus* can create a diverse set of heat-stable enterotoxins [12].

The number of semi-intensive and extensive smallholder dairy farms in Ethiopia has increased over time as a result of urbanization, rising human population, and rising incomes. However, these dairy farms’ management approaches remained consistent [13]. Furthermore, in traditional practice, the hygiene of the milker, the cow’s udder, the milking environment, and the milking equipment may be the primary cause of early milk contamination, and farmers do not adhere to normal hygienic practices throughout milk production. Ethiopia does not regularly inspect milk and milk products for hygiene [13–16].

Many foods promote *S. aureus* growth and toxin generation; however, milk, dairy products, and meats are popular carriers and are likely the most frequently associated with *Staphylococcus* food poisoning [16, 17]. The most common source of *S. aureus* infection in dairy products is utensils and milkers’ hands [8, 15].

Several studies from different parts of Ethiopia reported the widespread prevalence of *Staphylococcus aureus* (13.9-80%), which implies a lack of effective personal, environmental, and animal husbandry hygiene and sanitation practices [18–20]. Raw milk, milking equipment, and human hands may all contain resistant *Staphylococcus aureus*, putting consumers in danger. Furthermore, there has been little research into the prevalence of *Staphylococcus aureus* and risk factors contributing to milk contamination in smallholder dairy farms in the study area. Thus, we aim to assess hygiene and handling practices, estimate the prevalence of *S. aureus* in dairy cow milk and associated risk factors [19] (age, lactation stages, parity level, history of mastitis status, udder and leg hygiene, and management system), and antimicrobial susceptibility profiles of *Staphylococcus aureus* isolated from raw cow milk, and swabs in smallholder dairy farms in Meta District of Eastern Hararghe Zone, Oromia Reginal State, Ethiopia.

## Methods

### Description of the study area

**Fig. 1:**
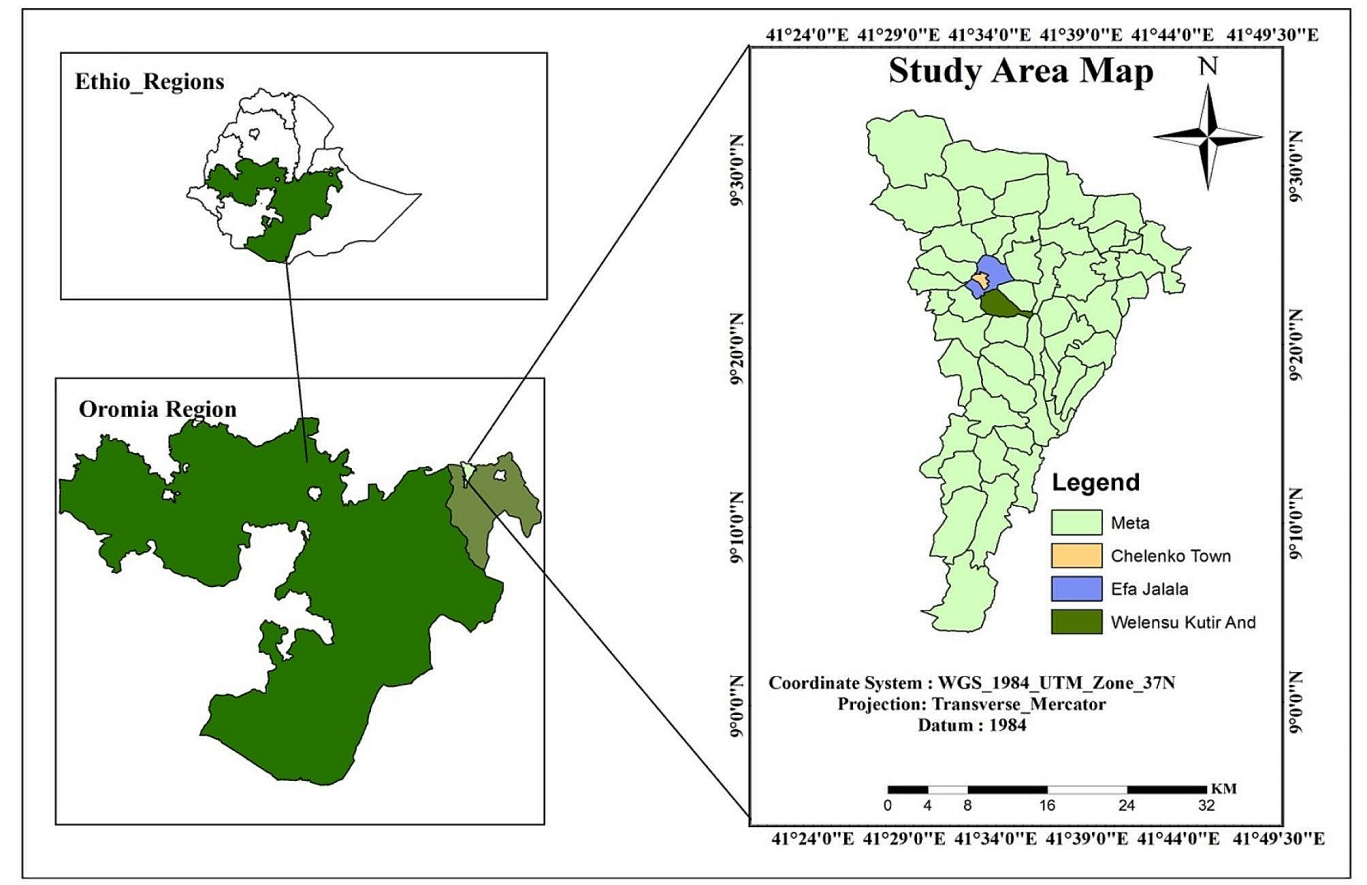
Map of the study area. (Source: ArcGIS, 2024)

The study was carried out in the Meta District of Eastern Oromia, Ethiopia (Fig. 1). Meta District is one of the 20 districts in East Hararghe, Oromia’s regional state. The district has the potential for animal resources and milk sheds, with a total population of 125,499: (49.5%) males and (51.5%) females. It is located 435 km east of Addis Ababa at 9°38” north latitude and 41°56” east longitude. The elevation of the area varies from 1400 to 2850 m above sea level. The temperature fluctuates from 17 °C to 27 °C, with a yearly average of 22 °C. The average yearly rainfall is 350–900 mm. July and August are the rainiest months. The farming approach for the study area is a mix of crops and livestock. According to the district’s Agricultural office, the livestock keeping and production methods are small and medium holder dairying, with a focus on indigenous cattle with a few cross breeds. These livestock owners also raise goats, sheep, and poultry. They graze moderately and widely as well as take advantage of the river and tap water. Grazing cattle and small ruminants separately is a widespread practice. Farmers retain these animals for a variety of purposes, including revenue, meat, milk, and draught power [21].

### Study animals

The study animals were healthy cross-bred lactating cows (Holstein Frisian × Indigenous local, Jersey × Indigenous local, and Borona × Indigenous local) from small and medium-sized dairy farms. They were exposed to a thorough yet semi-intensive management system. The study included 30 small and medium dairy farms chosen at random from a pool of 54 dairy farms based on owner willingness. The herd sizes on the chosen farms varied from 8 to 20 cattle, with three to sixteen lactating cows. In terms of management, 72 (61.5%) of the herds were intensively managed, with 45 (38.5%) being semi-intensive. The herd was classified as having at least ten cows. This is because farms with ten cows are small-scale farms in Ethiopia that operate as a sideline. Furthermore, small farms often lack sufficient feedstocks to last a year and are largely maintained by family members. Farms with more than 10 cows, on the other hand, are medium-scale semi-intensive farms that maintain feed reserves for the bulk of the year, employ paid labor, and are owned by dairy farmers. As a result, the herds were classified in this way simply to determine the impact of herd management modifications on *S. aureus* occurrence.

They wandered freely on natural pasture and shared similar water sources, but were milked in the morning and evening using roughage feeds like hay and crop wastes (such as maize stalks, wheat/barley straw, and other grain threshing remnants). The semi-intensively cared-for cattle grazed freely on grassland but were fed more in the morning and evening when milked. Every cow was hand-milked twice a day, in the morning and the evening.

Using farm owner information, the ages of the study dairy cows were determined and classified as young (3–5 years), adult (6–9 years), and old (> 9 years) [22, 23]. Parity was further categorized as few (1–2 calves), moderate (3–4 calves), or many (> 4 calves). Three lactation stages were used: early (> 3 months), medium (3–6 months), and late (> 6 months). Ruegg’s [24] four-point scale (1–4) was used to assess the hygiene of each cow’s udder and legs. An udder hygiene score (UHS) or leg hygiene score (LHS) of ‘1’ indicated that there was no contamination of the skin of the rear of the udder or the hind limb between the hock and coronary band; ‘2’ was slightly dirty (2–10% of the area covered in dirt); ‘3’ was moderately dirty (10–30% of the area covered in dirt); and ‘4’ was caked-on dirt (> 30% of these areas completely covered in dirty). The udder and leg hygiene of the sampled animals were graded as poor or good based on the accumulation of dirty sewage, muddy or appropriately cleansed animal parts, and a history of mastitis. In addition to animals, the study involved washing water, a milker’s hand, and a milking bucket [24].

### Study design and sample type

From December 2018 to August 2019, a cross-sectional study was carried out to estimate the prevalence of *Staphylococcus aureus* in raw cow milk and swabs of various contact surfaces, as well as to assess milk management practices. Furthermore, the antibiotic susceptibility profile of isolated *Staphylococcus aureus* was assessed using standard microbiology laboratory methods. The samples used included raw milk from a cow’s udder, as well as swabs from the milkers’ hands and milking equipment.

### Questionnaire survey

Face-to-face interviews with a structured questionnaire were used to collect data on key herd and animal-level characteristics that influence the prevalence of *Staphylococcus aureus* in dairy farms (Supplementary file). In addition, the questionnaire was validated through a systematic process to ensure reliability and validity. Initially, it was developed using relevant literature and expert interviews for content validity. Subject matter experts reviewed the draft for face validity, assessing clarity, relevance, and comprehensiveness. A pilot study with a small, representative sample identified ambiguous questions and evaluated internal consistency using Cronbach’s alpha. Poor-performing items were revised or removed. Exploratory factor analysis was conducted to confirm construct validity. After this rigorous validation process, the refined questionnaire was used in the main survey to collect reliable and valid data.

Similarly, the current survey considered the hygiene of the barn/milking environment, the hygiene of milking cows’ udders and milk handlers, the hygiene of milking equipment, with a focus on the hygiene of milking and milk handling practices, the utensils used for milking, milk storage, and milk uses. In addition, milk consumption patterns and awareness of the risk of zoonotic diseases related to raw cow milk consumption were explored. Similarly, the study considered cow-level factors such as lactation (age, parity, and stage), udder and leg hygiene, and mastitis history. During the interview, the questions were translated from English to Afan Oromo. The replies were then translated into English and included in the original format.

All questionnaire survey respondents were chosen based on their desire to participate; as a result, respondents from 30 farms were questioned about sanitary practice and public health relevance (Consumer at Farm Level) and recorded accordingly.

### Sample size determination and sampling techniques

Using the [25] formula, the sample size ‘n’ was calculated as follows:

$${\rm{N}} = \frac{{{{1.96}^2}({{\rm P}_{\exp }}(1 - {{\rm P}_{\exp }})}}{{{{\rm{d}}^2}}}$$

Where 1.96 = the value of Z at a 95% confidence interval,

d = desired absolute precision,

n = required sample size,

P_exp_=expected prevalence.

Accordingly, considering a 95% confidence interval, a 5% desired absolute precision, and an 8.3% previous prevalence [26], a minimum calculated sample size was 117. As a result, three peasant associations (3PAs) were intentionally chosen: Chelenko, Ifa Jalela, and Wallensu. Furthermore, eight, ten, and twelve smallholder dairy farms were carefully picked from each PAs, respectively; based on the number of dairy farm owners, milk production and accessibility, availability of one or more lactating animals, and dairy farm owners’ willingness. After assigning an identity code to each lactating animal, 117 lactating cows were chosen by simple random sampling methods. Likewise, 30 swab samples from the milkers’ hand and 30 swab samples from milking equipment were collected based on the number of workers, frequency of visits to the farm, and materials utilized. Finally, *S. aureus* was isolated and identified from 177 separate samples.

### Sample collection and transportation

A 25-ml raw cow milk sample from each of the 117 healthy lactating cows was collected aseptically using sterile universal bottles with screw caps [27]. Swab samples were obtained from the milkers’ hand and milking equipment before milking by wiping zigzag over above contact surfaces with wet sterile swabs in saline solution, which were subsequently maintained in sample bottles containing sterile physiological saline solution to avoid desiccation. All samples were promptly transported in an ice box to Haramaya University’s College of Veterinary Medicine Microbiology Laboratory and refrigerated (4^o^C) until examination. It took less than 24 h to isolate the bacterial species [28].

### Laboratory analysis

#### Isolation and identification of staphylococcus aureus

The pre-enriched milk samples were inoculated onto mannitol salt agar and incubated at 37 °C for 24 h. The presence of growth and a pH change in the media (from red to yellow) were considered confirmatory of *Staphylococcus* identification. Using the phenol red pH indicator, the acidic metabolic product of mannitol was identified. When *S. aureus* ferments mannitol, the medium becomes yellow. After 24 h of incubation, colonies that produced a faint or delayed yellow color were classified as *S. intermedius*, but colonies that produced no change in the media were identified as *S. hyicus* [29]. *S. aureus* was confirmed biochemically with the coagulase test. Suspected *S. aureus* colonies were placed in tubes containing 5 ml of brain heart infusion broth and incubated at 37^o^C for 20–24 h before being mixed with 0.3 ml of rehydrated rabbit plasma in small tubes and incubated at the same temperature. After 6 h, the tubes were checked for clotting [30].

The catalase test technique was utilized to identify suspicious colonies based on Gram’s reactivity and cellular shape. Gram-stained smears from typical colonies were recognized as *Staphylococcus* species and tested for catalase activity. Catalase-positive staphylococci colonies were then isolated and subcultured on mannitol salt agar before being incubated aerobically at 37 °C for 24 to 48 h. Coagulase tests were performed on staphylococci colonies that had become yellow on the media. To distinguish pathogenic staphylococci, the coagulase-positive staphylococci isolate was inoculated on purple base agar (containing 1% maltose) and aerobically incubated at 37 °C for 24–48 h. The identification was based on *S. aureus’s* rapid fermentation of maltose, which turned the medium and colonies yellow. *S. intermedius* produces a weak or delayed response, while *S. hyicus* does not ferment maltose [29]. Finally, the isolated *S. aureus* colonies were evaluated for antibiotic resistance.

### Antimicrobial susceptibility test

Antibiotic susceptibility patterns of *Staphylococcus aureus* isolates were examined using the disc diffusion method. Briefly, *S. aureus* isolates were suspended in 5 ml of sterile saline (0.85% NaCl) to match the 0.5 MacFarland turbidity standard. The suspensions were then swabbed across the whole surface of Mueller Hinton agar (Oxoid) with a sterile cotton swab and left on the bench to absorb excess moisture [31]. The contaminated surface was then covered with discs containing individual quantities of each antimicrobial agent (Oxoid, Basing Stoke, and UK) and incubated overnight at 37 °C. The clear zones of bacterial growth inhibition were measured in millimeters with a straight-line ruler. Growth inhibition zone sizes were categorized as susceptible, intermediate, or resistant [32]. Susceptibility testing was conducted using 10 drugs namely amoxicillin (AMX) (25 g), ampicillin (AM) (10 g), penicillin (10 g), tetracycline (TE) (30 g), erythromycin (ER) (15 g), streptomycin (10 g), vancomycin (30 g), sulphamethoxazole (30 g), cefoxitin (15 g), and kanamycin (30 g).

### Data management and analysis

The raw data were entered and coded in a Microsoft Excel spreadsheet 2016 before being analyzed using STATA^®^ version 14.0 statistical software (Stata Corp. College Station, USA). *Staphylococcus aureus* prevalence, antibiotic susceptibility test percentages, and questionnaire data proportions were calculated. The prevalence was used as an outcome variable in logistic regression analysis against the hypothesized risk factors’ explanatory variables (breed, sex, age, body condition, herd size, and history of mastitis). In univariable analysis, explanatory variables with a *p*-value ≤ 0.25 (maximum likelihood ratio test) were selected for multiple logistic regression analyses. The final multiple logistic regression models were created manually, using a forward stepwise selection approach. A confounder was defined as a variable that impacted the coefficient of the significant variables by more than 25%. Kruskal gamma statistics were used to analyze the predictors’ multicollinearity in the models, and variables with gamma values ranging from − 0.6 to + 0.6 were included in a multivariate logistic regression model. The final multivariate logistic regression models were used to compute the odds ratio (OR) and 95% confidence interval (CI) of the factors influencing the outcome variables. Significant differences were considered at a *p*-value < 0.05.

## Results

### Assessment of respondent on the concepts of hygienic practice of milk and its public health significance

A structured questionnaire survey of 30 smallholder farm owners at the farm level was used to assess the public health implications of *Staphylococcus aureus* and suspected sources of milk contamination. Consequently, 86.7% of the farmers cleaned the barn once every day, whereas 13.3% cleaned it twice a day. However, 80% did not wash cow udder or teat, while 56.7% and 30% washed their hands before and after milking. Also, 30% of dairy workers used detergent to clean their equipment before milking. All farmers used plastic containers for milking and storage. Moreover, the current survey revealed that 63.3% of dairy farmers drink raw milk. Only 36.7% of dairy farmers consumed boiled milk, while 73.3% were unaware of foodborne infections (Table 1).

**Table 1:** Hygienic practices and habits of handling milk in the study area (*n* = 30)

Variables		No of examined	No. positive	Prevalence (%)
**Hand washing before milking**	Yes	17	2	56.7
	No	13	11	43.3
**Hand washing between milking**	Yes	9	0	30
	No	21	13	70
**Udder washing before milking**	Yes	6	3	20
	No	24	10	80
**Udder washing after milking**	Yes	3	1	10
	No	27	12	90
**The detergent used to clean milk Equipment**	Yes	14	2	30
	No	16	11	70
**Milk storage**	Plastic	100	100	100
**Fumigation uses milk Equipment**	Yes	17	6	56.7
	No	13	7	43.3
**Milking order**	Sequential	4	2	13.3
	Random	26	11	86.7
**Milking mastitis cows**	Yes	13	10	43.3
	No	17	3	56.6
**Barn cleaning**	Once	26	13	86.7
	Twice	4	0	13.3
**Source of water**	River	19	9	63.3
	Tap	11	4	36.7
**Mixing milk of different cows**	Yes	22	12	73.3
	No	8	1	26.7
**Milk stayed before sold**	Up to 6	19	10	63.3
	More than 6	11	3	36.7
**Milk consumption**	Raw	19	12	63.3
	Boiled	11	1	36.7
**Acquiring illness**	Yes	7	7	23.3
	No	23	6	76.7
**Gastrointestinal truck disturbance-drinking raw milk**	Yes	12	12	40
	No	18	1	60
**Aware of foodborne infection**	Yes	9	1	26.7
	No	21	12	73.3

### Prevalence of *Staphylococcus aureus*

**Fig. 2:**
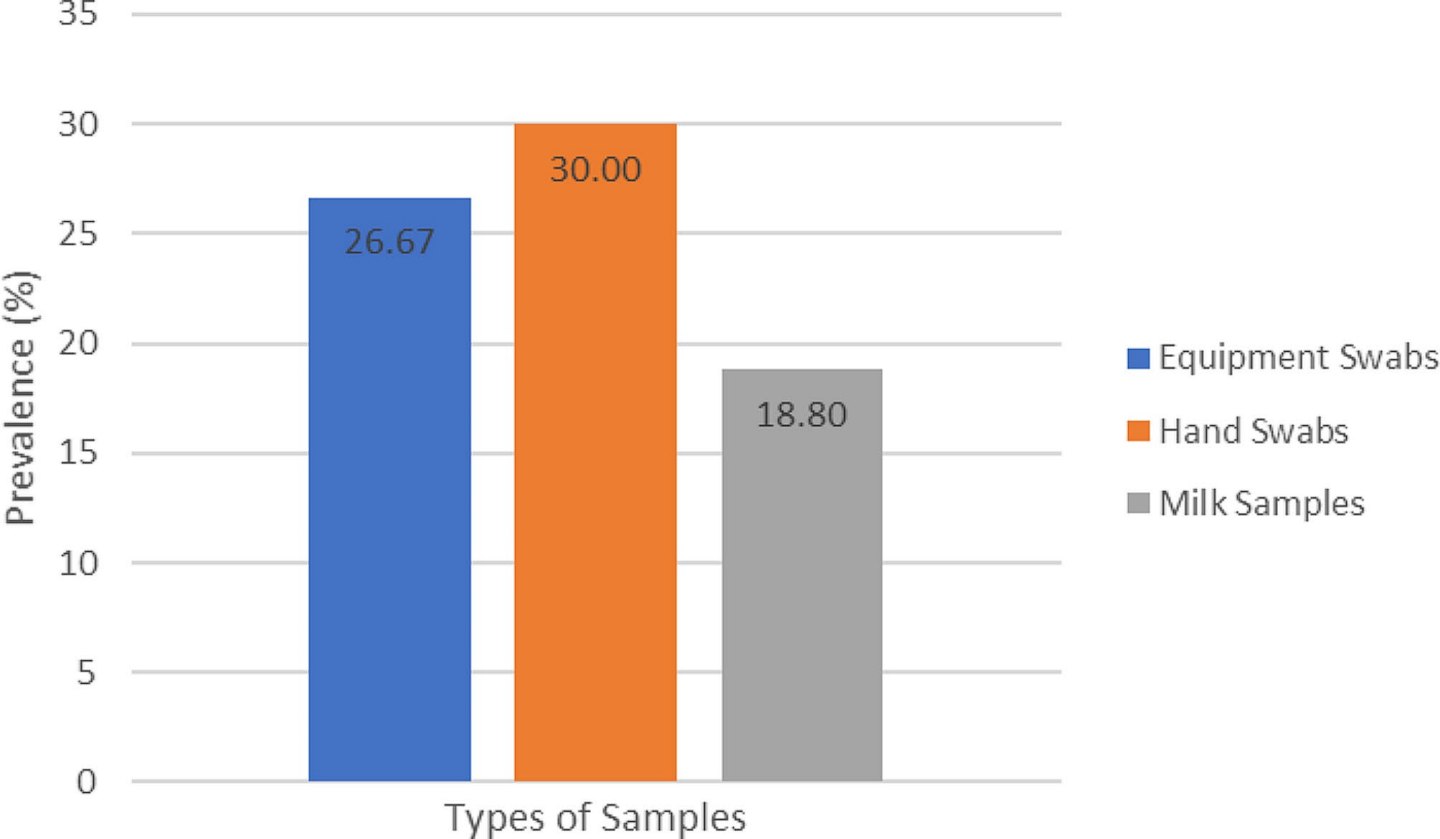
Samples-wise prevalence of *Staphylococcus aureus*

The current study found that 30% prevalence of *Staphylococcus aureus* in hand swabs with an overall prevalence of 12.42% (95%CI: 8.32–18.98) among tested samples (Fig. 2).

Old age, poor udder and leg hygiene, and mastitis-positive animals had the highest prevalence of *Staphylococcus aureus* at 36.84%, 30.76%, and 29.72%, respectively, compared to counterparts. *Staphylococcus aureus* prevalence by kebele, sexes, BCS, parity level, udder and leg hygiene, and mastitis history is shown in Table 2.

**Table 2:** The prevalence of *Staphylococcus aureus* with associated risk factors in the study area

Variable	Categories	No. examined	No. Positive	%	95%CI
**PAs**	Chellencko	35	9	25.71	13.72–42.95
	Ifa Jalela	54	8	14.81	7.48–27.22
	Wallensu	28	5	17.85	74.37–37.03
**Management System**	Extensive	30	8	26.66	13.66–45.51
	Sem-intensive	87	14	16.09	9.68–25.53
**Age**	Old	19	7	36.84	18.14–60.55
	Adult	45	5	11.11	46.10-24.42
	Young	53	10	18.86	10.33–31.93
**Parity level**	Mild	59	11	18.64	10.51–30.88
	Few	30	3	10.00	3.15–27.46
	Many	28	8	28.57	14.67–48.18
**Lactation level**	Early	35	9	16.00	8.09–29.18
	Mid	50	8	25.71	13.72–42.95
	Late	32	5	15.62	65.00-33.03
**Udder and leg hygiene**	Good	91	14	15.38	9.25–24.48
	Poor	26	8	30.76	15.85–51.18
**History of mastitis status**	Yes	37	11	29.72	17.04–4655
	No	80	11	13.75	77.03–23.34
Total		117	22	12.42	8.32–18.98

PAs = Peasant Association, CI = Confidence Interval; %= Prevalence

Univariable logistic regression analysis revealed that age and history of mastitis in lactating cows were deemed risk factors for the prevalence of *Staphylococcus aureus* and exhibited statistically significant variation (*p* < 0.05) (Table 3).

**Table 3:** Univariable logistic regression analysis of *Staphylococcus aureus* with associated risk factors

Variable	Categories	No. Positive	%	OR	95% CI for OR	*p*-value
**PAs**	Chellencko	6	17.14	1.59	0.46–5.44	0.458
	Ifa Jalela	5	92.59	0.8	0.23–2.72	0.721
	Wallensu	11	39.28	Ref.	-	-
**Management System**	Extensive	8	26.66	2.43	0.91–6.48	0.074
	Sem-intensive	14	16.09	Ref.	-	-
**Age**	Old	7	36.84	4.66	1.25–17.40	0.022
	Young	10	18.86	1.86	0.58–5.91	0.293
	Adult	5	11.11	Ref.	-	-
**Parity level**	Few	3	10.00	0.48	0.12–1.89	0.297
	Many	8	28.57	1.74	0.61–4.98	0.298
	Mild	11	18.64		Ref.-	-
**Lactation level**	Early	9	16.00	1.86	0.55–6.32	0.314
	Mid	8	25.71	1.02	0.30–3.47	0.964
	Late	5	15.62	Ref.	-	-
**Udder and leg hygiene**	Poor	8	30.76	2.44	0.89–6.70	0.082
	Good	14	15.38	Ref.	-	-
**History of mastitis status**	Yes	11	29.72	2.65	1.02–6.85	0.044
	No	11	13.75	Ref.	-	-

PAs = Peasant Association, OR = Odds Ratio; Ref = Referent category; CI = Confidence Interval; %= Prevalence

**Fig. 3:**
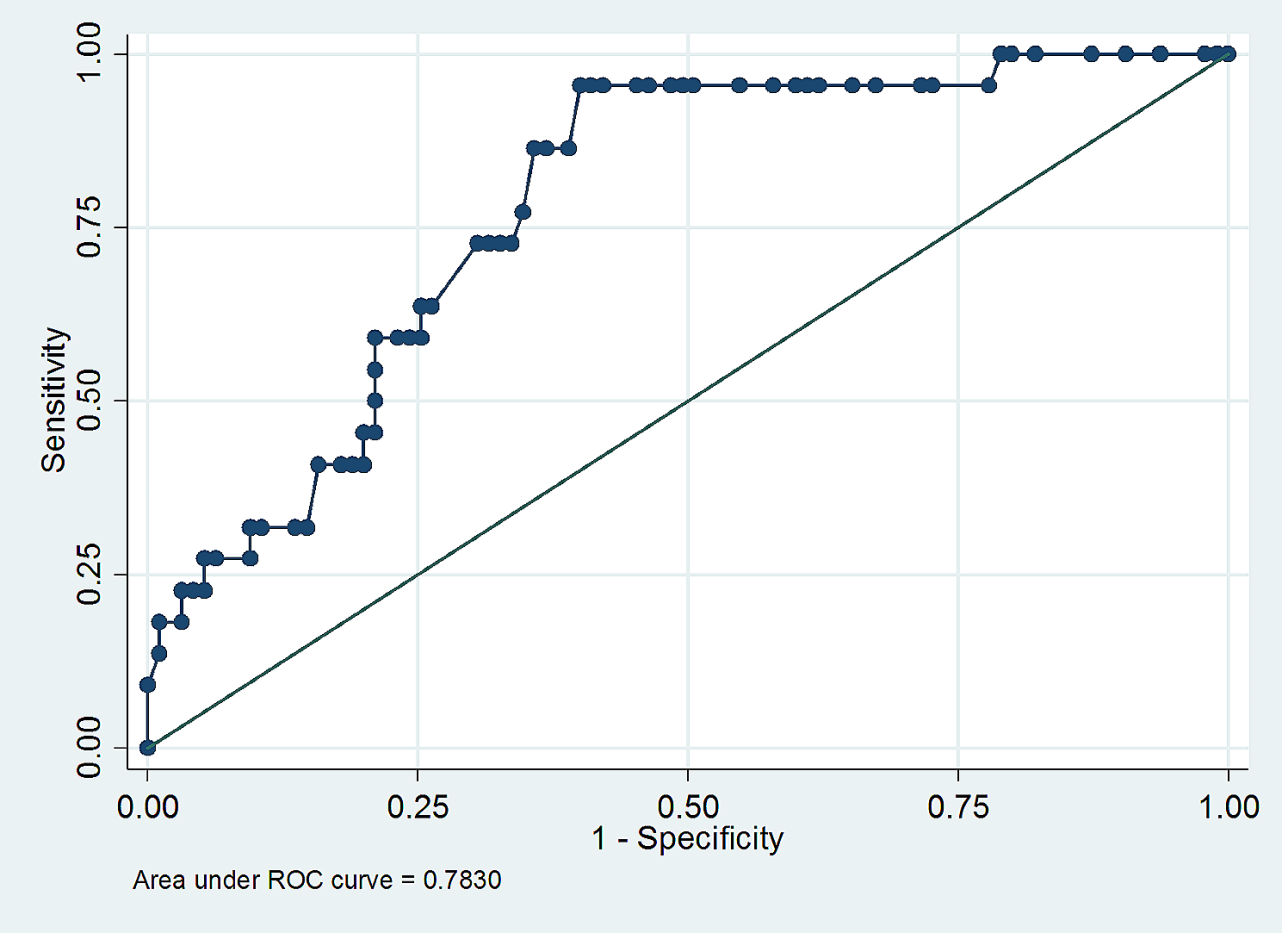
Multicollinearity test

Following collinearity testing, all variables with *p* ≤ 0.25 in the initial analysis (management systems, age, udder, and leg hygiene, and history of mastitis status) were subjected to stepwise forward selection of multivariable logistic regression analysis. In the final model, age and mastitis history were significant predictors of *Staphylococcus aureus* (*p* < 0.05). Likewise, old and mastitis positive animals were eight (OR: 8.40; 95%CI: 1.68–41.89) and four (OR: 4.33; 95%CI: 1.37–13.66) times more likely to be infected by *S. aureus* than adult, and mastitis negative animal. Moreover, the Hosmer-Lemeshow goodness-of-fit test suggested that the model fit the data (χ2 = 16.20; *p* = 0.7493) and multicollinearity was found not to violate the assumption (AUC = 78.30%) (Table 4; Fig. 3).

**Table 4:** Multivariable logistic regression analysis of *Staphylococcus aureus* with associated risk factors

Variable	Categories	No. Positive	%	OR	95% CI for OR	*p*-value
**Age**	Old	7	36.84	8.40	1.68–41.89	0.009
	Young	10	18.86	2.31	0.63–8.49	0.205
	Adult	5	11.11	Ref.	-	-
**History of mastitis status**	Yes	11	29.72	4.33	1.37–13.66	0.012
	No	11	13.75	Ref.	-	-

### Antimicrobial susceptibility test of *Staphylococcus aureus* isolates

In this study, antimicrobial drugs were tested on 22 *Staphylococcus aureus* isolates. Ten (10) drugs important to veterinary and human health were chosen, and resistant patterns were examined using the disc diffusion technique. Penicillin, Tetracycline, and Sulphamethezole exhibited resistance rates of 97.6%, 69.2%, and 66.7%, respectively. In contrast, the isolates were shown to be susceptible to kanamycin, streptomycin, vancomycin, and cefotixin at 84.6%, 71.8%, 64%, and 58.8%, respectively (Table 5).

**Table 5:** Antimicrobial-resistance test of *Staphylococcus aureus* in the study area (*n* = 39)

Antimicrobial	Susceptible No. (%)	Intermediate No. (%)	Resistance No. (%)
**AMP**	16(41.02)	4(10.3)	19 (48.7)
**SXT**	11(28.2)	2(5.13)	26(66.7)
**TE**	12 (30.8)	0	27(69.2)
**CEFTIX**	23(58.8)	2(5.2)	14(35.9)
**STREPTO**	28(71.8)	6(15.4)	5(12.8)
**VANCO**	25(64.0)	4(10.6)	10(25.6)
**PE**	1(2.6)	0	38(97.4)
**AMX**	22(56.4)	0	17(43.6)
**KANAMY**	33(84.6)	5(12.8)	1(2.56)
**ER**	6(15.4)	21(53.8)	12(30.8)

Note: Amoxicillin = AMX, Ampicillin = AMP), Penicillin = PE; Tetracycline = TE, Erythromycin = ER, CEFTIX = Cefotixin, STREPTO = Streptomycin, VANCO = vancomycin, SXT = sulphamethoxazole, KANAMY = kanamycin

## Discussion

Ethiopia is a developing country, and dairy farming is an essential component of the agricultural production system. Milk and milk products are in great demand due to the country’s constantly increasing population and urbanization. Although milk is crucial for consumer nutrition and producer revenue in Eastern Ethiopia, data are scarce on the evaluation of hygienic practices and bacteriological contamination of raw cow milk [33].

### Hygienic practices of milk and its public health significance

The current study found that 80% of the farmers did not wash the cow udder or teat, but 56.0% and 30% cleaned their hands before and after milking, respectively. They did not use antiseptic remedies to clean their hands before milking. In contrast, 70% of respondents did not use detergent to clean dairy equipment before milking, while 46.7% did. All farmers used plastic containers for milking and storage. Dry towels and freezers are not utilized. This shows that additional involvement is required to create awareness for milking personnel or farmers on the significance of hygienic milk handling and husbandry methods. Moreover, in the majority of smallholder dairy producers, insufficient dairy house cleaning methods and dirty settings, as well as milkers’ poor personal hygiene, are sources of pathogens for *S. aureus* and other diseases [34].

This study revealed that the majority of workers involved in milking activities lacked access to hygienic milking settings and equipment. All of these variables make milk prone to microbial infection at home. Hand milking in a dirty animal home, not washing the cow udder and/or teats before milking, irresponsible milking personnel, and not washing hands before milking have all been implicated as potential sources of microbial contamination in milk. Except for a few urban cowkeepers, most barns were not built to acceptable standards of design. Yoseph et al. [35] and Yitaye et al. [36] reported similar effects on dairy producers in Ethiopia’s northwestern highlands. During the field inspection, it was observed that the barns were not designed to allow for farm waste drainage, resulting in the soiling of dairy cows and milk contamination.

The current survey demonstrated that 86.7% of respondents clean the barn every day by simply removing dung, while 13.3% clean twice a day. This is in line with reports of Zelalem [37], in the Ethiopian highlands, over 87% of respondents cleaned their barns every day, with 9% cleaning only three times each week. Cows’ teats and udders grow dirty when sleeping in stalls or loitering in muddy barnyards. Microorganisms have been identified in considerable quantities in soiled bedding [38]. After cleaning, milking was done in the same spot. Even though most dairy cow owners keep their barn floors clean, dry, and pleasant bedding environment is essential for preventing the spread of hazardous germs. Exposed teat end practices, as well as wet and muddy pens, raise the possibility of *S. aureus* occurrence and milk contamination [24].

Many barns had dirty living conditions; which implies animal shelters have received insufficient attention. This has the potential to impact the quality of milk and milk products produced and processed. To produce milk and milk products of acceptable quality, a clean and sanitary living environment is required [39].

In the current study, all of the smallholder dairy farms in the study area milk by hand, which means they do not utilize a machine. Similarly, 43.3%, 70%, and 80% of responders did not wash their hands before, after milking, and the cow udder (before milking), respectively. These findings were consistent with previous reports of [40–43], who reported that 75.8% of farmers in various localities did not clean cow udders before milking. Most dairy farm owners fail to fully clean and dry the cow’s udder and teat with clean water. In contrast to the current study, Haile et al. [44] revealed that 82.5% of small-scale farm-owning households in Hawassa City do pre-milking udder washing. Cleaning the udder before milking is necessary to remove apparent debris and bacteria from its outer surface.

Furthermore, a cow’s udder and teat must be cleaned before milking because they may come into direct touch with the ground, urine, excrement, and feed refusals while resting, thereby contaminating the milk. Not only should the udder be cleaned before milking, but using low-quality water for cleanliness (hands and milk equipment), failing to cover milk after milking, and storing milk at room temperature for an extended period can all introduce contaminations into the milk. However, pre-milking udder preparation and good milk handling techniques are crucial in preventing *S. aureus* infection on the farm [45, 46]. Thus, producers should make udder washing a regular practice to avoid contamination and provide high-quality milk.

Plastic containers were used (100%) for milking, storing, collecting, and transporting at smallholder dairy farms in the study area. Similarly, [47] stated that all respondents in and around Jigjiga City of Somali Region perform manual milking, with more than 60% of the interviewed families using plastic jars as milking and transportation equipment. Plastic containers, which were commonly used, are difficult to clean, particularly at the bottom and inner corners, where milk residue might cling. Microorganisms can quickly accumulate in potentially nutritious milk residues from storage containers, contaminating the milk when it is later used. This is in line with the report of [48]. Plastic containers are simple to scrape and provide hiding spots for germs during cleaning and sanitization; also, they are poor heat conductors, resulting in bacterial contamination of milk [49]. The majority of respondents in the study area washed their milk utensils. However, the cleaning is ineffective, and the utensils are not completely dry. Thus, milk contamination can occur when surfaces, such as dirty milking equipment and hands, come into contact with milk.

Farmers have limited access to clean water for cleaning milking equipment, udders, hands, and drinking. However, the river and tape waters used for washing may be of poor quality, contributing to the area’s low milk quality. As a result, it is critical to heat-treat river water and clean tap water. Good hygienic measures (clean milking equipment, hand cleansing, udder washing, and use of heat-treated water) are required during milking and handling before distribution to customers or processors [50].

In the current study, 63.3% of the respondents drink raw cow milk, 40% have GIT disturbances as a result of raw cow milk consumption, and 73.3% are unaware of milk-borne diseases. This is consistent with the findings of Ayele et al. [20], which found that 64% of respondents were uninformed of the risks of milk-borne disease associated with raw milk consumption. Besides, the incidence of GIT disturbance related to raw cow milk consumption was acknowledged; ingestion of raw milk without treatment may pose a public health risk because of its low safety and quality. As a result of this practice, consumers of milk-borne diseases are exposed to several risks [51, 52]. Similarly, despite livestock managers’ warnings about the risk of zoonotic infections and milk-borne diseases, the general populace continues to drink cow raw milk [53, 54].

Shirima et al. [53] detected several zoonotic ailments in small-holder dairy farms. Therefore, more public health education is required at all stages of the food supply chain (farmers, transporters, and consumers) to protect the public from animal-related health issues [53]. Furthermore, poor hygiene may result in low milk safety and quality, significantly reducing consumer demand. This is in line with [55], pathogenic microorganisms from the teat canal, an infected udder, or environmental pollutants from improper milking, handling, and storage can all reduce milk quality and safety.

### Prevalence of *Staphylococcus aureus*

The current study revealed 12.42% (95%CI: 8.32–18.98) of *Staphylococcus aureus* isolates and is consistent with studies conducted by Lencho [19], who reported 13.9% at Ambo and Guder town, Abebe et al. [56], who reported 15.5% at Addis Ababa, Fikru, [18], who reported 17.2% at Addis Ababa, and Eyasu, [57], who reported 17.85% at Arsi Negele town. This could be due to animal health specialists teaching good cleanliness habits and boosting awareness among stakeholders in the studied area.

Whereas the current finding disagrees with Mokonnen et al. [26], Ayele et al. [20], Abunna et al. [58], Tessema and Tsegaye [59], Abera et al. [60], Abera et al. [23], Tasew et al. [47], Wubete, [9], and Bedada and Hiko, [61], who reported 8% at Debreziet, 19.6% at Sebeta, 21.1% at Addis Ababa, 21.2% at Alage ATVET College Dairy Farm, Ethiopia, 28.1% in Shashemene, 42.1% in Adama, 26.6% in kombolcha, 27% at Addis Ababa, and 39.1%, *S. aureus* isolates at Asella, respectively. This variance could be attributed to sample sizes, husbandry practices, and dairy farmers’ awareness.

*Staphylococcus aureus* was isolated at a rate of 30% (9/30) from the milkers’ hands and 26.7% (8/30) from milking equipment swabs. This shows the milk handlers and milk equipment could be potential sources of *S. aureus* contamination in milk. The isolation rate from the milkers’ hand and milking equipment was equivalent to and lower than in the current study based on two sampling points reported by Ayele et al. [20], who found a prevalence of 32% and 11.1% in Sebeta, respectively. Furthermore, the rate of isolation from milkers’ hands corresponded to the prevalence rates reported by Andrade [62] and Tondo et al. [63], which were 35.7% and 35.2%, respectively. This could be because staphylococci are common organisms that at least half of the population carries in their nasal passages and throat and can contaminate by coughing or sneezing [64].

The current finding of *S. aureus* in milking bucket swabs is higher than that reported by Abunna et al. [65], who reported 0% in pooled bucket swabs at Asella and Lencho [19], who reported 9% in milking bucket swabs at Ambo and Guder town. The variation in the prevalence of *Staphylococcus aureus* isolates could be attributed to the milkers’ hygiene and equipment.

The current study found a statistically significant link between age groups (*p* < 0.05), with high prevalence recorded in the older, young, and adult age groups, respectively, at 36.84%, 18.86%, and 11.11%. This finding is similar to those undertaken by Girma et al. [18] in the Holeta area and Workineh et al. [66] in and around Bahir Dar. In the current study, the higher occurrence in older cows could be attributed to larger teats and more relaxed sphincter muscles, which allow infectious agents to enter and develop more easily in the cows’ udder. Furthermore, because milk is produced in high quantities over a lengthy period, older cows with different parity levels are more susceptible to udder contamination during the milking process. As a result of the strain, the teat canals may widen and allow germs to enter [67, 68].

The current study revealed a statistically negligible link among parity categories (*p* > 0.05), with high prevalence recorded in a few, moderate, and many parity cows, respectively, at 28.57%, 18.64%, and 10.00%. Erskine [69] claims that primiparous cows have more efficient defense systems than multiparous cows. When the parity number rises, it could be due to excessive contamination of the udder and milk during the milking process. Also, no statistically significant difference (*p* > 0.05) was observed in the lactation stage, with a prevalence of 15.62%, 16.00%, and 25.71% in late, early, and medium lactation stages, respectively. This is consistent with the findings of Abera et al. [23] in Adama and Lencho [19] in Ambo and Guder. Cows in early lactation were far less sensitive to microorganisms than cows in mid-lactation. This could be related to differences in neutrophils in the mammary gland in newly calved cows, as well as increased oxidative stress and impaired antioxidant defense systems during early lactation. Also, Belayneh et al. [70] found a greater prevalence of *S. aureus* in the late stages of lactations, whereas Mukriya et al. [71] found a considerably higher prevalence in the mid-stage in Kenya. The differences in the influence of lactation phases observed in different studies could be attributed to differences in the age, parity, and breed of the animals studied.

The status of cow udders and leg hygiene were also identified as risk factors for *S. aureus* prevalence. According to the udder and leg hygiene score, all of the cows evaluated had moderately to extremely unclean udders and legs, and pathogen detection increased significantly as dirtiness rose. Cows’ udders and legs were dirty due to poor sanitation on small-scale dairy farms. About 30.76% of the samples were deemed unclean and tested positive for *S. aureus*. This demonstrates a lack of waste drainage houses/shelters, as well as a buildup of dung and urine. In addition, a significant association has been reported between poor udder cleanliness and an increased risk of *S. aureus* [72].

In general, the high prevalence of *S. aureus* in this study could be attributed to a lack of post-milking teat dipping, a failure to cull chronically infected cows, a lack of dry cow therapy, and dairy herds’ preference for hand milking. *S. aureus* and other pathogenic bacteria are usually found on the udder or teat surface of infected cows, and they are the principal source of infection between uninfected and infected udder quarters, especially during milking. Milkers in all observed herds wash their hands before milking, but only for the first cow. As a result, infectious bacteria could easily spread from infected to uninfected udder quarters or animals through milkers’ hands. Antibiotic therapy has an exceedingly low cure rate for *S. aureus* infections during lactation, and many infected animals develop chronic infections and must be culled [46, 72]. Unfortunately, in the study area, none of the dairy farmers use chronically infected animal culling, dry cow therapy, or post-milking teat washing, making the environment conducive to the organism’s establishment in dairy herds.

### Antimicrobial susceptibility test

Antimicrobial susceptibility tests revealed the presence of *S. aureus* antimicrobial resistance. The occurrence of antibiotic-resistant *S. aureus* isolates could be related to indiscriminate antimicrobial usage, self-medication, and prophylactic administration of a subtherapeutic dose of antimicrobials to animals, as well as a lack of updating of long-used drug classes [72].

Antibiotics used in veterinary and human health were evaluated for overall activity against *S. aureus* isolates taken from a sample. The disc diffusion technique was used to screen 22 *S. aureus* isolates for various antibiotics. The resistance pattern varied among tens of drugs. The resistance rates for penicillin, tetracycline, and sulphamethoxazole were 97.6%, 69.2%, and 66.7%, respectively. This is consistent with the findings of [56], who discovered an antimicrobial resistance pattern of *S. aureus* to Penicillin of 87.2%, although it contradicts Haftay et al. [72] in the case of Tetracycline (0%). This could be attributable to the ability of *S. aureus* strains to modify their resistance behavior to previously exposed antimicrobials [73]. A new CLSI study [74] confirms *Staphylococcus aureus’s* significant tetracycline resistance (78.9%). This is not surprising given that penicillin G and tetracycline are the two most commonly used antimicrobials in Ethiopian veterinary practice for infection treatment. Furthermore, penicillin resistance is plasmatic, which means that it rapidly spreads to other strains [56]. Similarly, Daka et al. [75] reported that 67.9% of *S. aureus* isolates from milk were resistant to penicillin G. Furthermore, this high level of resistance was induced by the isolate generating a penicillin enzyme (a kind of -lactamase) that destroyed penicillin’s beta-lactam ring.

Tetracycline resistance is primarily plasmid-mediated and inducible in *Staphylococcus aureus*. Tetracycline accumulation is reduced by the acquisition of an energy-dependent efflux channel or lower influx, whereas tetracycline access to the ribosome (site of action) is reduced by the acquisition of ribosome-protected proteins and enzyme inactivation [76]. Resistance to one tetracycline frequently results in resistance to the others. Tetracycline was initially demonstrated to be highly efficient against *Staphylococcus aureus*; however, resistance has recently emerged and has become a therapeutic restriction [77]. In contrast, the isolate strains were found to be susceptible to Kanamycin, Streptomycin, Vancomycin, and Cefoxitin at 84.6%, 71.8%, 64%, and 58.8%, respectively.

The high resistance pattern observed in this study against Penicillin, Tetracycline, Sulphamethezole, and, to a lesser extent, Amoxicillin (43.6%) and Ampicillin (48.7%) is most likely due to selective pressure caused by uncontrolled and inappropriate use of these drugs in a veterinary clinic, study farms, and the country as a whole. Because *Staphylococcus aureus* is a normal flora member and thus utilized as a possible indication for resistance development in humans and animals, the absence of an antibiotic usage policy and the availability of other antibiotics in the country contribute to this [66]. Antibiotic usage causes pathogenic bacteria and flora bacteria to develop resistance strains. Historically, pathogenic bacteria attracted the most attention; but, more recently, the significance of commensal organisms as a reservoir or vehicle for spreading resistance genes to more dangerous pathogenic bacteria has been proposed [75].

Furthermore, the high level of antibiotic resistance among *Staphylococcus aureus* isolates could be attributed to a self-prescription policy, comparatively cheaper antibiotic consumption, a lack of reliance on laboratory guidance and adequate antibiotic doses, and indiscriminate antibiotic use in animal husbandry practices. Antibiotics are widely available in Ethiopia without a prescription from a qualified veterinary medical practitioner. This is the leading cause of antibiotic abuse. Resistance that develops in flora bacteria may be transferred to other bacteria and infect humans through direct or indirect channels, particularly resistant strains associated with livestock antibiotics (tetracycline, erythromycin, streptomycin [66, 75].

The in vitro disc sensitivity test revealed that Kanamycin is the most effective antibiotic, followed by Streptomycin, Vancomycin, and Cefoxitin, which is consistent with a report in Adama by Belayneh et al. [70] and Abera et al. [23], who found (90%) and (86.1%) susceptibility to Kanamycin, respectively. Because they are not commonly utilized in veterinary clinics, these antimicrobials may be less resistant. Similarly, Katakweba et al. [77] claimed that antimicrobial resistance is almost invariably the outcome of recurrent therapeutic or indiscriminate use of antibiotics.

Previous studies by Belayneh et al. [70] revealed that amoxicillin reduced *S. aureus* strains in Adama by 62%. Drug resistance poses a public health risk because food-borne epidemics can be difficult to cure, and multi-drug resistant *S. aureus* in the food chain acts as a reservoir for communicable resistant genes [72].

## Conclusion

This study revealed that the smallholder dairy farmers have a low level of hygienic practices and habits of handling milk in the study area. This reduces the safety of raw cow milk and milk products for consumers. The overall prevalence of *S. aureus* was 12.42%, whereas 18.8% in raw milk, 30% in milkers’ hand swabs, and 26.7% in milking equipment swabs. Only age and history of mastitis were potential risk factors for the *S. aureus* prevalence in milk. Furthermore, the isolates were resistant to penicillin G at 97.4% and tetracycline at 69.2%. Conversely, they were susceptible to kanamycin, streptomycin, vancomycin, and cefotaxime, at 84.6%, 71.8%, 64%, and 58.8%, respectively. The study identified antimicrobial-resistant patterns of *S. aureus* in raw cow milk, inadequate milk processing practices, and raw milk consumption habits. Thus, awareness should be created for the smallholder dairy farmers about hygienic milk handling practices, milk-borne diseases, and rational uses of drugs. Also, antimicrobial sensitivity tests should be examined before use.

### Electronic supplementary material

Below is the link to the electronic supplementary material.

Supplementary Material 1

## Data Availability

Data is available on request from the corresponding author.

## Abbreviations

BAP: Blood Agar Plates
BPW: Buffered Peptone Water
CHP: Centre for Health Protection
CLSI: Clinical Laboratory Standard Institute
CNS: Coagulase-Negative Staphylococcus
CPS: Coagulase Positive Staphylococcus
FBD: Food Borne Disease
GIT: Gastro Intestinal Tract
H_2_O_2_: Hydrogen peroxide
IDF: International Dairy Federation
MSA: Mannitol salt agar
MWAO: Meta Wereda Agricultural Office
NaCl: Sodium Chloride
NAP: Nutrient Agar Plates
NCCLS: National Committee for Clinical Laboratory Standard
OF: Oxidation-Fermentation
PA: Peasant Association
PAB: Purple Agar Base
PBPs: Penicillin Binding Proteins
PH: Power of hydrogen
SFP: Staphylococcus food poisoning
SSSS: Staphylococcal Scalded Skin Syndrome
TNase: Thermo Nuclease

## References

[1] World Health Organization. Food safety risk analysis: A guide for national food safety authorities. https://apps.who.int/iris/bitstream/handle/10665/43718/9789251056042_eng.pdf 17891885

[2] Havelaar AH, Kirk MD, Torgerson PR, Gibb HJ, Hald T, Lake RJ, Praet N, Bellinger DC, De Silva NR, Gargouri N, Speybroeck N. World Health Organization global estimates and regional comparisons of the burden of foodborne disease in 2010. PLoS Med. 2015;12(12):e1001923. 10.1371/journal.pmed.1001923. 26633896 10.1371/journal.pmed.1001923 PMC4668832

[3] Kirk MD, Pires SM, Black RE, Caipo M, Crump JA, Devleesschauwer B, Döpfer D, Fazil A, Fischer-Walker CL, Hald T, Hall AJ. World Health Organization estimates of the global and regional disease burden of 22 foodborne bacterial, protozoal, and viral diseases, 2010: a data synthesis. PLoS Med. 2015;12(12):e1001921. 10.1371/journal.pmed.1001921. 26633831 10.1371/journal.pmed.1001921 PMC4668831

[4] Pandey G, Voskuil GC. Manual on milk safety, quality, and hygiene Golden Valley Agricultural Research Trust. Zambia. 2011;52:5.

[5] Pal M. Public health hazards due to consumption of raw milk. Ethiop Herald. 2012 Mar;14:10.

[6] Bertu WJ, Dapar M, Gusi AM, Ngulukun SS, Leo S, Jwander LD. Prevalence of brucella antibodies in marketed milk in Jos and environs. Afr J Food Sci. 2010;4(2):62–4. https://academicjournals.org/journal/AJFS/article-full-text-pdf/620540721664.

[7] Pal M, Jadhav VJ. Microbial contamination of various Indian milk products. Beverage Food World. 2013;40(12):43–4.

[8] Girma K, Tilahun Z, Haimanot D. Review on milk safety with emphasis on its public health. World J Dairy Food Sci. 2014;9(2):166–83. 10.5829/idosi.wjdfs.2014.9.2.85184. 10.5829/idosi.wjdfs.2014.9.2.85184

[9] Wubete A. Bacteriological quality of bovine milk in smallholder dairy farms in Debre Zeit, Ethiopia. Vet Res. 2004;4:34–7.

[10] Singh PA, Prakash A. Prevalence of coagulase-positive pathogenic *Staphylococcus aureus* in milk and milk products collected from the unorganized sector of Agra. Acta Agriculturae Slov. 2010;96(1):37–41. http://aas.bf.uni-lj.si/zootehnika/96-2010/PDF/96-2010-1-37-41.pdf.

[11] Giangiacomo R. Milk testing, quality control, hygiene and safety.

[12] Larsen HD, Sloth KH, Elsberg C, Enevoldsen C, Pedersen LH, Eriksen NH, Aarestrup FM, Jensen NE. The dynamics of *Staphylococcus aureus* intramammary infection in nine Danish dairy herds. Vet Microbiol. 2000;71(1–2):89–101. 10.1016/S0378-1135(99)00161-3. 10665537 10.1016/S0378-1135(99)00161-3

[13] Gemechu T. Microbial Quality and Associated Public Health Hazards of Raw Cow’s Milk Produced and Marketed in Ethiopia: A review.http://www.nbmedicine.org

[14] Reta MA, Addis AH. Microbiological quality assessment of raw and pasteurized milk. Int J Food Sci Microbiol. 2015;2(6):087–91.

[15] Gemechu T, Beyene F, Eshetu M. Handling practices and microbial quality of raw cow’s milk produced and marketed in Shashemene Town, Southern Ethiopia. Int J Agric Soil Sci. 2014;2:153–62.

[16] Yilma Z, Loiseau G, Faye B. Manufacturing efficiencies and microbial properties of butter and ayib-ethiopian cottage cheese. Livest Res Rural Dev. 2007;19(7).

[17] Smith K, Peter K, Daniela H, Melchior S. Foodborne pathogenic microorganisms and natural toxins. Food drug Administration center food safety. Appl Nutr. 2007;10:119–50.

[18] Fikru G. Epidemiology and its drug resistance in cattle, Food Chains and Human in Central Ethiopia. Addis Ababa, Ethiopia: Addis Ababa University; 2014.

[19] Lencho M. Identification and antimicrobial susceptibility profiles of *Staphylococcus* species isolated from raw milk, swabs of udders, milking utensils, and milkers hands in smallholder and dairy farms in Ambo and Guder town. Addis Ababa, Ethiopia: Addis Ababa University; 2015.

[20] Ayele Y, Gutema FD, Edao BM, Girma R, Tufa TB, Beyene TJ, Tadesse F, Geloye M, Beyi AF. Assessment of *Staphylococcus aureus* along the milk value chain and its public health importance in Sebeta, central Oromia, Ethiopia. BMC Microbiol. 2017;17(1):1–7. 28655298 10.1186/s12866-017-1048-9 PMC5488358

[21] MWAO (Meta Wereda Agricultural and Natural Recourse Office). The Annual plan document for 2017. Unpublished.

[22] Asefa I, Legabo E, Wolde T, Fesseha H. Study on Salmonella Isolates from Fresh Milk of Dairy Cows in Selected Districts of Wolaita Zone, Southern Ethiopia. International Journal of Microbiology. 2023;2023. 10.1155/2023/6837797 PMC9981291 36875709

[23] Abera M, Demie B, Aragaw K, Regassa F, Regassa A. Isolation and identification of *Staphylococcus aureus* from bovine mastitic milk and their drug resistance patterns in Adama town. Ethiopia J Veterinary Med Anim Health. 2010;2(3):29–34.

[24] Ruegg PL. The role of hygiene in efficient milking. WCDS Adv Dairy Technol. 2006;18:285–93.

[25] Thrusfield M. Veterinary epidemiology. Wiley; 2018 Apr. p. 30.

[26] Mekonnen A, Mahindra P, Moses N. Isolation and identification of *Staphylococcus* species from raw bovine milk in Debre Zeit. Ethiopia Medwell J. 2011;4(2):45–9.

[27] Abera T, Legesse Y, Mummed B, Urga B. Bacteriological quality of raw camel milk along the market value chain in Fafen Zone, Ethiopian Somali regional state. BMC Res Notes. 2016;9(1):1–6. 27230392 10.1186/s13104-016-2088-1 PMC4880963

[28] Tola A, Ofodile LN, Beyene F. Microbial quality and chemical composition of raw whole milk from Horro cattle in East Wollega, Ethiopia. Ethiop J Educ Sci. 2007;3(1):1–0.

[29] Quinn PJ, Markey BK, Carter ME, Donnelly WJ, Leonard FC. Veterinary microbiology and microbial disease. Blackwell Science; 2002.

[30] Bogere P, Baluka SA. Microbiological quality of meat at the abattoir and butchery levels in Kampala City, Uganda. Internet J Food Saf. 2014;16(2014):29–35.

[31] Hudzicki J. Kirby-Bauer disk diffusion susceptibility test protocol. Am Soc Microbiol. 2009;15:55–63.

[32] Wayne PA, Clinical, Standards L. Institute; 2007. Performance standards for antimicrobial susceptibility testing. CLSI document M100-S17. 2005.

[33] Godefay B, Molla B. Bacteriological quality of raw cow’s milk from four dairy farms and a milk collection center in and around Addis Ababa. Berl Munch Tierarztl Wochenschr. 2000;113(7–8):276–8. https://europepmc.org/article/med/10994252. 10994252

[34] Nanu E, Latha C, Sunil B. Quality assurance and public health safety of raw milk at the production point. Am J Food Technol. 2007;2(3):145–52. 10.3923/ajft.2007.145.152

[35] Mekasha Y, Tagegn A, Yami A, Umunna NN. Evaluation of the general farm characteristics and dairy herd structure in urban and peri-urban dairy production systems in Addis Ababa milk shed. Challenges and Opportunities of Livestock Marketing in Ethiopia; 2003. p. 139.

[36] Ayenew YA, Wurzinger M, Tegegne A, Zollitsch W. Handling, processing and marketing of milk in the north western Ethiopian highlands. Livest Res Rural Dev. 2009;21(7):97.

[37] Yilma Z. Microbial properties of Ethiopian marketed milk and milk products and associated critical points of contamination: an epidemiological perspective. Epidemiol Insights. 2012;20:297.

[38] Murphy SC, Boor KJ. Trouble-shooting sources and causes of high bacteria counts in raw milk. Dairy, Food and Environmental Sanitation. 2000;20(8):606 – 11.

[39] Asaminew T. Production, handling, traditional processing practices, and quality of milk in Bahir Dar milk shed Area, Ethiopia. Ethiopia: Haramaya University; 2007.

[40] Derese T. *Present situation of urban and peri-urban milk production and quality of raw milk produced in West Shewa Zone, Oromia Region* (Doctoral dissertation, M. Sc. Thesis. Haramaya University, Ethiopia).

[41] Bejano S. A study on prevalence, public health significance, and Associated risk factors of *bacillus cereus* on bovine raw milk in and around Assosa district, in household dairy farms of the Benishangul Gumuz regional state. Western Ethiopia, 2014.

[42] Swai ES, Schoonman L. Microbial quality and associated health risks of raw milk marketed in the Tanga region of Tanzania. Asian Pac J Trop Biomed. 2011;1(3):217–22. 23569762 10.1016/S2221-1691(11)60030-0 PMC3609189

[43] Shija F. *Assessment of milk handling practices and bacterial contaminations along the dairy value chain in Lushoto and Handeni districts, Tanzania* (Doctoral dissertation). 10.1002/vms3.71034 PMC13270394 42299863

[44] Welearegay H, Yilma Z, Tekle-Giorgis Y. Hygienic practices and microbiological quality of raw milk produced under different farm sizes in Hawassa, southern Ethiopia. Agricultural Res Rev. 2012;1(4):1132–42.

[45] Michelle A. *Staphylococcus aureus* Mastitis UK Veterinary Diagnostic Laboratory, and Jeffrey Bewley, Animal and Food sciences. Volume 40546. Lexington, KY: University of Kentucky College of Agriculture; 2011.

[46] Sears PM, McCarthy KK. Management and treatment of staphylococcal mastitis. Veterinary Clinics: Food Anim Pract. 2003;19(1):171–85. 10.1016/s0749-0720(02)00079-8 12682941

[47] Tassew A, Negash M, Demeke A, Feleke A, Tesfaye B, Sisay T. Isolation, identification and drug resistance patterns of methicillin-resistant *Staphylococcus aureus* from mastitic cows milk from selected dairy farms in and around Kombolcha, Ethiopia. J Veterinary Med Anim Health. 2016;8(1):1–0. 10.5897/JVMAH2015.0422

[48] Bukuku JN. *Awareness of health risks as a result of consumption of raw milk in Arusha City and Meru District, Tanzania* (Doctoral dissertation, Sokoine University of Agriculture).

[49] Omoe K, Hu DL, Takahashi-Omoe H, Nakane A, Shinagawa K. Comprehensive analysis of classical and newly described staphylococcal superantigenic toxin genes in *Staphylococcus aureus* isolates. FEMS Microbiol Lett. 2005;246(2):191–8. 15899405 10.1016/j.femsle.2005.04.007

[50] Getachew F. A Review of the small-scale dairy sector in Ethiopia. FAO prevention of food losses program. milk and milk products, post-harvest losses and food safety in sub-Saharan Africa and Near East. 2003.

[51] Desissa FA. *Quantitative risk assessment of consuming milk contaminated with Staphylococcus aureus in Debre-Zeit* (Doctoral dissertation, Addis Ababa University).

[52] Lues JF, De Beer H, Jacoby A, Jansen KE, Shale K. Microbial quality of milk, produced by small scale farmers in a peri-urban area in South Africa. Afr J Microbiol Res. 2010;4(17):1823–30.

[53] Shirima GM, Fitzpatrick J, Cleaveland S, Kambarage DM, Kazwala RR, Kunda J, French NP. Participatory survey on zoonotic diseases affecting livestock keeping communities in Tanzania. J Anim Vet Adv. 2003;2:253–8.

[54] Mosalagae D, Pfukenyi DM, Matope G. Milk producers’ awareness of milk-borne zoonoses in selected smallholder and commercial dairy farms of Zimbabwe. Trop Anim Health Prod. 2011;43:733–9. 21120606 10.1007/s11250-010-9761-5

[55] Matofari JW, Shitandi A, Shalo PL, Nanua NJ, Younan M. A survey of Salmonella enterica contamination of camel milk in Kenya. Afr J Microbiol Res. 2007;1(4):46–50.

[56] Mekuria A, Asrat D, Woldeamanuel Y, Tefera G. Identification and antimicrobial susceptibility of *Staphylococcus aureus* isolated from milk samples of dairy cows and nasal swabs of farm workers in selected dairy farms around Addis Ababa, Ethiopia. Afr J Microbiol Res. 2013;7(27):3501–10.

[57] Tsige E. Investigation of *Staphylococcus aureus* along the milk value chain and assessment of. Its public health significance in Arsi Negelle town, Ethiopia. Vol. Masters. Addis Ababa, Ethiopia: Addis Ababa University; 2018.

[58] Abunna F, Fufa G, Megersa B, Regassa A. Bovine mastitis: prevalence, risk factors and bacterial isolation in small-holder dairy farms in Addis Ababa City, Ethiopia. Global Vet. 2013;10(6):647–52.

[59] Tessema D, Tsegaye S. Study on the prevalence and distribution of *Staphylococcus aureus* in raw cow milk originated from AlageAtvet College Dairy Farm, Ethiopia. J Nutr Food Sci. 2017;7(2):2–5.

[60] Abera M, Habte T, Aragaw K, Asmare K, Sheferaw D. Major causes of mastitis and associated risk factors in smallholder dairy farms in and around Hawassa, Southern Ethiopia. Trop Anim Health Prod. 2012;44:1175–9. 22231019 10.1007/s11250-011-0055-3

[61] Bedada BA, Hiko A. Mastitis and antimicrobial susceptibility test at Asella, Oromia Regional State, Ethiopia. J Microbiol Antimicrobials. 2011;3(9):228–32.

[62] Andrade GP, Zelante F. Simultaneous occurrence of enterotoxigenic *Staphylococcus aureus* on the hands and in the mouth and stools of asymptomatic carriers. Rev Saúde Pública. 1989;23:277–84. 2631181 10.1590/S0034-89101989000400002

[63] Tondo EC, Guimarães MM, Henriques JA, Ayub MA. Assessing and analyzing contamination of a dairy products processing plant by *Staphylococcus aureus* using antibiotic resistance and PFGE. Can J Microbiol. 2000;46(12):1108–14. 11142400 10.1139/w00-111

[64] Argaw S, Addis M. A review on staphylococcal food poisoning. Food Sci Qual Manage. 2015;40(2015):59–72.

[65] Abunna F, Abraham T, Gizaw F, Beyene T, Feyisa A, Ayana D, Mamo B, Duguma R. *Staphylococcus*: isolation, identification and antimicrobial resistance in dairy cattle farms, municipal abattoir and personnel in and around Asella, Ethiopia. J Veterinary Sci Technol. 2016;7(6):1–7. 10.4172/2157-7579.1000383

[66] Workineh S, Bayleyegn M, Mekonnen H, Potgieter LN. Prevalence and etiology of mastitis in cows from two major Ethiopian dairies. Trop Anim Health Prod. 2002;34:19–25. 11887418 10.1023/A:1013729626377

[67] Madut NA, Gadir AE, Jalii IM. Host determinants of bovine mastitis in the semi-intensive production system of Khartoum state, Sudan. J Cell Anim Biology. 2009;3(5):071–7.

[68] Constable PD, Hinchcliff KW, Done SH, Grünberg W. Veterinary medicine: a textbook of the diseases of cattle, horses, sheep, pigs, and goats. Elsevier Health Sciences; 2016 Oct. p. 25.

[69] Erskine RJ. Mastitis control in dairy herds. Herd Health Food Animal Production Medicine. Philadelphia, Penn: WB Saunders Co; 2001. pp. 397–433.

[70] Belayneh R, Belihu K, Wubete A. Dairy cows mastitis survey in Adama town. Ethiopia J Veterinary Med Anim Health. 2013;5(10):281–07.

[71] Lakew M, Tolosa T, Tigre W. Prevalence and major bacterial causes of bovine mastitis in Asella, South Eastern Ethiopia. Trop Anim Health Prod. 2009;41:1525–30. 19333772 10.1007/s11250-009-9343-6

[72] Haftay A, Geberemedhin H, Belay A, Goytom E, Kidane W. Antimicrobial resistance profile of *Staphylococcus aureus* isolated from raw cow milk and fresh fruit juice in Mekelle, Tigray, Ethiopia. J Veterinary Med Anim Health. 2018;10(4):106–13. 10.5897/JVMAH2017.0664

[73] Quinn PJ, Markey BK, Leonard FC, Hartigan P, Fanning S, Fitzpatrick E. Veterinary microbiology and microbial disease. Volume 7. Wiley; 2011 Oct.

[74] CLSI (Clinical and Laboratory Standards Institute). Investigation and control of Vancomycin-intermediate and resistant *Staphylococcus aureus*. Wayne, PA: A guidebook for health departments and infection control personnel; 2008.

[75] Daka D, G/silassie S, Yihdego D. Antibiotic-resistance *Staphylococcus aureus* isolated from cow’s milk in the Hawassa area, South Ethiopia. Ann Clin Microbiol Antimicrob. 2012;11:1–6. 25927182 10.1186/1476-0711-11-26 PMC3549789

[76] Chopra I, Roberts M. Tetracycline antibiotics: mode of action, applications, molecular biology, and epidemiology of bacterial resistance. Microbiol Mol Biol Rev. 2001;65(2):232–60. 11381101 10.1128/MMBR.65.2.232-260.2001 PMC99026

[77] Katakweba AA, Mtambo MM, Olsen JE, Muhairwa AP. Awareness of human health risks associated with the use of antibiotics among livestock keepers and factors that contribute to the selection of antibiotic-resistant bacteria within livestock in Tanzania. Livest Res Rural Dev. 2012;24(10):170.

