# Supplementary material for: Identification and antimicrobial susceptibility profiles of Staphylococcus species isolated from raw cow milk, and swabs in smallholder dairy farms in Meta district, Eastern Ethiopia
Source: BMC Microbiol. 2024 Aug 1;24:284. doi: 10.1186/s12866-024-03439-6 (PMC11292862; doi:10.1186/s12866-024-03439-6)
Supplement: Supplementary file 1 — Supplementary Material 1 [file 12866_2024_3439_MOESM1_ESM.doc]

Questionnaire I: Information related to individual animal history, hygienic practice, and its public health significance.

- **General information:**

Dairy Farm name: ____________________________Owners Name: ______________ Address__________________ Date of sample Collection_____________________

1. What is the name of the farms?
2. Herd size:

2. What type of management system is used? 1. Semi-intensive 2. Extensive

2. **Animal information (sampled cattle):**

1. What age of the sampled cow? Answer(s)…………….

2. What parity level of the sampled cow? Answer(s)…………….

3. What lactation stage of the sampled cow? Answer(s)…………….

4. Udder and leg hygiene of sampled cow? --------------------

5. History of mastitis of sampled cow? Answer(s)…………….

Questionnaire **II**: Information on the hygienic practice of milk and its public health significance

Date: ____________ Address (farm name): __________ Sex ____________

1. Is hand washing practiced before milking? 1. Yes 2. No

2. Is hand washing practiced between milking? 1. Yes 2. No

3. Is that udder washed and dried before milking? 1. Yes 2. No

4. Is the udder washed and dried after milking? 1. Yes 2. No

5. Is milking mastitis cows? 1. Yes 2. No

6. Is that antiseptic used for cleaning during milking? 1. Yes 2. No

7. What kind of milking utensils do you use? a. plastic b. Stainless steel c.others

8. What kind of storage containers do you use? 1. Plastic 2. Stainless steel 3. others

9. Do you use detergents for washing of milk containers? a. yes b. no

10. How long is milk stored after milking at home before sold?

11 . How long does it stay at home? a. <2 hr b. 2-6 hr c. 6-12hr

12. Where do you store unsold milk? 1. with no cooling 2. In the refrigerator

13. Is there any means other than cooling for the preservation of milk? a. yes b. no

14 . Milking orders? a. Sequentially b. Randomly

15. Fumigation to use milking Equipment? a. yes b. no

16. Cleaning barn? a. once a day b. Twice a day c.3 times a week

17. Source of water farm use? a. river b. tap water c.Others

18. Habit of milk consumption? a. raw b. boiled c.others

19. Do you mix fresh and leftover milk for consumption? a. yes. no

20. Do you know any health risks associated with raw milk consumption? a. yes b. no

21. Do you pool/bulk milk from different sources/cows? a. yes b. no

22. Do you know any GIT disturbance associated with drinking raw milk? a. yes b. no

23. Did you suffer from milk-borne infections? a. yes b. no

24. Do you know any risk of Staphylococci disease cause? a. yes b. no
